# Supplementary material for: Amelioration of Obesity in Mice Fed a High-Fat Diet with Uronic Acid–Rich Polysaccharides Derived from Tremella fuciformis
Source: Polymers (Basel). 2022 Apr 8;14(8):1514. doi: 10.3390/polym14081514 (PMC9029567; doi:10.3390/polym14081514)
Supplement: Supplementary file 1 [file polymers-14-01514-s001.zip › polymers-1640592-supplementary.pdf]

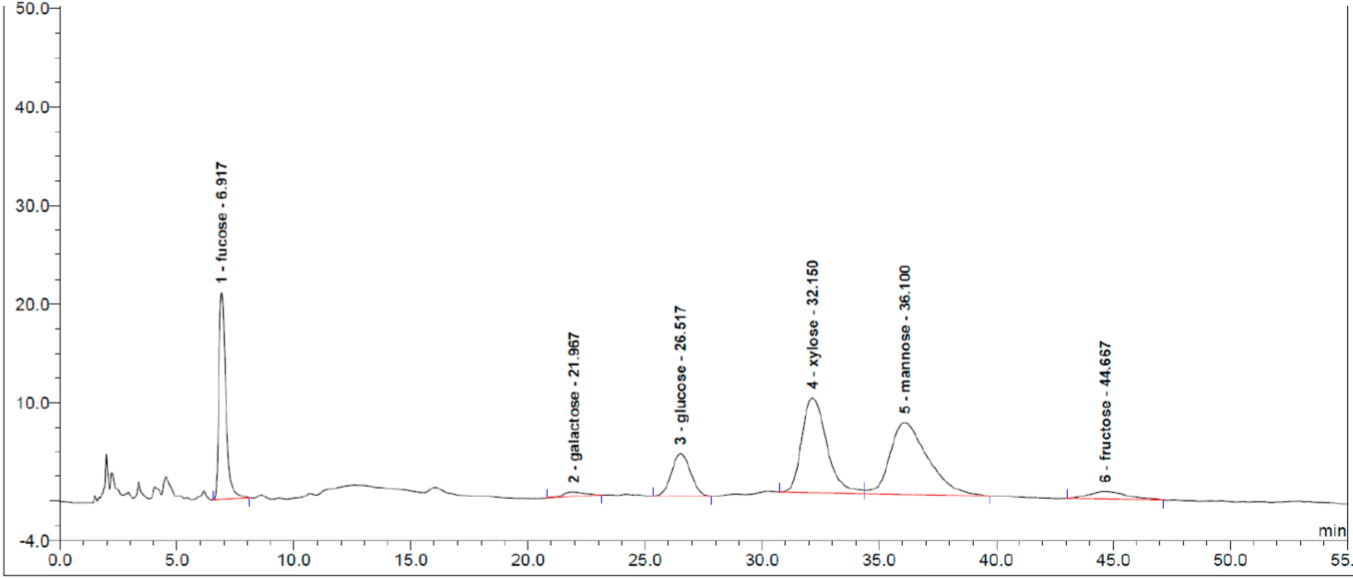

Supplement Figure S1. Monosaccharide composition analysis by HPAEC−PAD. TFPS was hydrolysis by 2M TFA.
